# The characteristics of patient safety culture in Japan, Taiwan and the United States

**DOI:** 10.1186/1472-6963-13-20

**Published:** 2013-01-14

**Authors:** Shigeru Fujita, Kanako Seto, Shinya Ito, Yinghui Wu, Chiu-Chin Huang, Tomonori Hasegawa

**Affiliations:** 1 Department of Social medicine, Faculty of Medicine, Toho University, Tokyo, Japan; 2 Department of Senior Citizen Service Management, Ming-Hsin University of Science and Technology, Hsinchu, Taiwan

**Keywords:** Safety, Organizational culture, Safety management, Attitude of health personnel, Patient safety culture

## Abstract

**Background:**

Quality and safety issues are receiving growing attention. Patient safety culture (PSC) plays an important role in patient safety. The characteristics of PSC in various countries, each with a different set of values, have not been determined sufficiently. The aim of this study is to investigate the characteristics of PSC in Japan, Taiwan and the U.S.

**Methods:**

A cross-sectional survey was conducted in Japan and Taiwan using the Hospital Survey on PSC (HSOPS) questionnaire developed by the U.S. Agency for Healthcare Research and Quality (AHRQ). Data from Japan and Taiwan were also compared with the U.S. “2010 HSOPS Comparative Database” provided by AHRQ.

**Results:**

Valid response rates in Japan, Taiwan and the U.S. were 66.5% (6,963/10,466), 85.7% (10,019/11,692) and 35.2% (291,341/827,424), respectively. The proportion of respondents with some experience of event reporting during the past 12 months was highest in Japan. In general, U.S. healthcare workers were likely to evaluate their PSC higher than that in Japan or Taiwan. The attitude of continuous improvement in Japan and event reporting of near misses in Taiwan were rated as low. In the U.S., staffing was rated as high.

**Conclusions:**

The results suggest that PSC varies among different countries, and the cultural setting of each country should be given special consideration in the development of effective intervention plans to improve PSC. Additional investigations with improved methodology and a common protocol are required to accurately compare PSCs among countries.

## Background

Quality and safety issues in healthcare continue to receive a growing amount of attention. The role of patient safety culture (PSC) is regarded as important for patient safety. A positive PSC is considered to guide many discretionary behaviours of healthcare professionals towards viewing patient safety as one of their highest priorities [1]. PSC is defined as the product of individual and group values, attitudes, perceptions, competencies, and patterns of behaviour, which determine the commitment to and the style and proficiency of an organization's health and safety management [2]. Although several measurement tools were developed, the Hospital Survey on PSC (HSOPS), which was developed by the U.S. Agency for Healthcare Research and Quality (AHRQ), is used widely all over the world [3-7]. Previous studies that used subjects from a country reported internal consistency and construct validity [3-7]. HSOPS was also used to assess the effect of interventions for improving patient safety such as encouraging teamwork within hospital units [8-11]. Although HSOPS subjectively evaluates safety-related responses, it did report on the relationship between clinical outcomes and the PSC values measured by HSOPS [11]. PSC may differ according to professions, units, hospitals, countries, and others. Although multiple studies have referred to PSC results obtained in other studies, few statistical analyses have been conducted [5-7,12,13]. Chen et al. (2010) used statistical analysis to compare Taiwanese and U.S. PSC, and highlighted some of the characteristics of Taiwanese healthcare workers [7]. However, the subjects originated from teaching hospitals, and the sample size appeared insufficient to understand the characteristics of Taiwanese healthcare workers. For international comparisons of PSC, diverging values should be considered because PSC could be affected by race, religion, healthcare system, and other similar variables. The PSC characteristics from countries with different values have not been explored sufficiently. We hypothesized that PSC characteristics were similar among East Asian countries, whose cultures were closely related, and different from those of western countries, whose cultures include heterogeneous customs such as interpersonal relationships. The aim of this study is to investigate and compare the PSC characteristics of Japan, Taiwan, and the U.S.

## Methods

For comparing PSC, we conducted a questionnaire-based, anonymous, and self-administered cross-sectional survey to healthcare workers in Japan and Taiwan. The HSOPS questionnaire was used to measure PSC in each country. Japanese and Taiwanese data were also compared with the U.S. ‘2010 HSOPS Comparative Database’, which was provided by AHRQ. The survey period and recruitment strategy of hospitals and respondents in each country differed from one another; details are listed in Table 1[2,14,15]. Only acute care hospitals participated in this study because the questionnaire was developed only for this type of hospital.

**Table 1:** Materials and response rates

	**Japan**	**Taiwan**	**U.S.**
Recruiting strategy			
Hospitals	Voluntary participation^§^	Stratified sampling & Voluntary participation	Voluntary participation^§^
Respondents in the hospital	All healthcare workers	A part of the healthcare workers^†^	All healthcare workers or a part of the healthcare workers^††^
Period of survey	Jan 2009–Jan 2010	Jul 2007–Aug 2008	Jan 2006–Jun 2009
Number of participated hospitals			
<300 beds	3	23	702
300–499 beds	7	13	117
≥500 beds	4	38	65
Total	14	74	884
Number of the subjects			
Distribution	10,466	11,692	827,424
Recovery	8,192 (78.3%)	10,289 (88.0%)	337,862 (40.8%)
Valid data^‡^	6,963 (66.5%)	10,019 (85.7%)	291,341 (35.2%)

^§^Hospitals were not selected randomly and participation was voluntary.

^¶^Ten hospitals participated voluntarily and 64 were selected randomly.

^†^Depending on the number of beds in each hospital, 10–300 questionnaires were distributed.

^††^The questionnaires were distributed to all staff at 637 hospitals and to some staff at 247 hospitals.

^‡^Incomplete questionnaires were excluded from the analysis, and included surveys with less than an entire section completed, surveys with fewer than half of the total items completed, or surveys with identical responses to every item.

### Measures

HSOPS had 51 questions and assessed healthcare worker opinions or attitudes about patient safety issues, errors in hospital settings, or event reporting. It included 42 items that measure 12 sub-dimensions of PSC, 2 items regarding PSC outcome measures, and 6 items regarding background information of the respondent. Each item used a Likert scale of 5-point response options of degree of agreement (1: strongly disagree to 5: strongly agree) or frequency (1: never to 5: always). The questions related to the PSC outcome measures included both a Patient Safety Grade that asked respondents to provide an overall grade on patient safety in their respective departments (1: failing to 5: excellent) and a Number of Events that asked respondents to provide the number of events they reported during the previous 12 months (1: no event to 5: 21 events or more). The requested background information included years in current profession, hours of work per week, and direct interaction with patients [2,15].

The sub-dimensions of PSC consisted of (1) Frequency of Events Reported, (2) Overall Perceptions of Safety, (3) Supervisor/Manager Expectations and Actions Promoting Safety, (4) Organizational Learning/Continuous Improvement, (5) Teamwork within Hospital Units, (6) Communication Openness, (7) Feedback and Communication about Error, (8) Non-punitive Response to Error, (9) Staffing, (10) Hospital Management Support for Patient Safety, (11) Teamwork Across Hospital Units, and (12) Hospital Handoffs and Transitions.

For each sub-dimension, the proportion of positive responses (percent positive score) was calculated for every respondent based on the AHRQ instructions, and it ranged from 0 to 1; higher scores indicated a more positive PSC. The total score of each sub-dimension, which was the sum of the item scores within each sub-dimension, was also calculated and ranged from 3 to 20; higher scores indicated a positive PSC. Although the percent positive scores were recommended by AHRQ for use in analysis, the total scores may have contained more respondent information because total scores reflect the 5-point response to each item. Results with greater accuracy may have been achieved if the total scores were used for the comparisons [2,15].

The HSOPS questionnaire was translated into Japanese and Mandarin. For the Mandarin version, backward translation was performed to confirm that the accuracy of the questionnaire was maintained. In contrast, the Japanese version was translated by a panel comprising a bilingual English–Japanese translators and specialists in patient safety, and backward translation was not used; other experts in safety culture verified the accuracy of the translation [14].

### Data analysis

To estimate internal consistency, we used Cronbach’s *α* to calculate values with each sub-dimension of each country. The percent positive scores, total scores, and other items belonging to a particular sub-dimension were compared among Japan, Taiwan, and the U.S. using Tukey’s honestly significantly different (HSD) test. In addition, the impact of each effect (Cohen’s *d*) was calculated by dividing the difference in score means of 2 countries by the pooled standard deviation. In our study, an absolute value of Cohen’s *d* of at least 0.5 indicated that the difference was significant. Chi-square tests were used to compare categorical variables. Statistical analyses were conducted using SPSS 19.0 (SPSS Inc.; Chicago, IL, USA).

### Ethical concerns

According to the Ethical Guidelines for Epidemiological Research, which was drawn up by the Japanese government, the approval of the ethics committee was not required because it was an anonymous and self-administered survey with no intervention or mental anguish [16]. In Taiwan, the survey was approved by Institutional Human subject Ethic Committee of National Chung Cheng University.

## Results

The response rates for each country are listed in Table 1. Respondent characteristics are shown in Table 2. Among the respondents, a significantly lower proportion of nurses were included in the U.S. (36.6%) compared with Japan (58.1%, P < 0.01) and Taiwan (57.0%, P < 0.01). The proportion of U.S. respondents who worked fewer than 40 hours per week (40.4%) was significantly higher than that of either Japan (23.6%, P < 0.01) or Taiwan (14.1%, P < 0.01). The proportion in Japan who reported at least 1 adverse event or near-miss event during the previous 12 months (64.0%) was significantly higher than that in Taiwan (48.0%, P < 0.01) and the U.S. (46.6%, P < 0.01). With regard to Patient Safety Grade, the proportion of U.S. respondents who answered ‘excellent’ or ‘very good’ (70.8%) was significantly higher than that in Japan (44.6%, P < 0.01) or Taiwan (37.7%, P < 0.01).

**Table 2:** Respondent characteristics

		**Japan**		**Taiwan**		**U.S.**	
		N	(%)	N	(%)	N	(%)
Occupation	Nurse^‡^	4,047	(58.1)	5,714	(57.0)	106,710	(36.6)
	Patient Care Assistant/Hospital Aide/Care Partner	228	(3.3)	148	(1.5)	16,529	(5.7)
	Physician^†^	597	(8.5)	961	(9.7)	11,881	(4.1)
	Pharmacist	171	(2.5)	448	(4.5)	5,203	(1.8)
	Dietician	221	(3.2)	31	(0.3)	1,739	(0.6)
	Unit Assistant/Clerk/Secretary	237	(3.4)	166	(1.7)	17,982	(6.2)
	Respiratory Therapist	0	(0.0)	38	(0.4)	6,710	(2.3)
	Physical, Occupational, or Speech Therapist	133	(1.9)	253	(2.5)	8,109	(2.8)
	Technician (e.g. EKG, Lab, Radiology)	543	(7.8)	718	(7.2)	32,097	(11.0)
	Administration/Management	562	(8.1)	1,029	(10.3)	20,292	(7.0)
	Others	187	(2.7)	277	(2.8)	54,028	(18.5)
	No answer	37	(0.5)	236	(2.4)	10,061	(3.5)
Years in current profession	Less than 1 year	605	(8.7)	896	(8.9)	17,977	(6.2)
	1–5 years	2,142	(30.8)	3,687	(36.8)	72,504	(24.9)
	6–10 years	1,366	(19.6)	2,643	(26.4)	49,500	(17.0)
	11–15 years	891	(12.8)	1,413	(14.1)	36,127	(12.4)
	16–20 years	617	(8.9)	695	(6.9)	32,174	(11.0)
	21 years or more	1,005	(14.4)	508	(5.1)	68,607	(23.5)
	No answer	337	(4.8)	177	(1.8)	14,452	(5.0)
Working hours in hospital	Less than 20 hours per week	439	(6.3)	113	(1.1)	12,948	(4.4)
	20–39 hours per week	1,206	(17.3)	1,303	(13.0)	104,958	(36.0)
	40–59 hours per week	3,936	(56.5)	7,380	(73.7)	141,250	(48.5)
	60–79 hours per week	513	(7.4)	728	(7.3)	12,136	(4.2)
	80–99 hours per week	80	(1.1)	190	(1.9)	7,445	(2.6)
	100 hours per week or more	13	(0.2)	83	(0.8)	343	(0.1)
	No answer	776	(11.1)	222	(2.2)	12,261	(4.2)
Number of events reported in the past 12 months	No event reports	2,428	(34.9)	4,894	(48.8)	147,892	(50.8)
	1–2 event reports	2,609	(37.5)	3,201	(31.9)	80,018	(27.5)
	3–5 event reports	1,362	(19.6)	1,091	(10.9)	35,716	(12.3)
	6–10 event reports	377	(5.4)	349	(3.5)	12,552	(4.3)
	11–20 event reports	73	(1.0)	101	(1.0)	4,587	(1.6)
	21 event reports or more	35	(0.5)	71	(0.7)	3,021	(1.0)
	No answer	79	(1.1)	312	(3.1)	7,555	(2.6)
Patient safety grade^††^	Failing	66	(0.9)	37	(0.4)	2,139	(0.7)
	Poor	479	(6.9)	442	(4.4)	12,614	(4.3)
	Acceptable	3,015	(43.3)	4,662	(46.5)	62,801	(21.6)
	Very Good	2,803	(40.3)	3,345	(33.4)	130,707	(44.9)
	Excellent	299	(4.3)	432	(4.3)	75,348	(25.9)
	No answer	301	(4.3)	1,101	(11.0)	7,732	(2.7)
Total		6,963		10,019		291,341	

^‡^Registered Nurse, Licensed Vocational Nurse, or Licensed Practical Nurse.

^†^Attending Physician, Staff Physician, Resident Physician, Physician in Training, Physician Assistant, or Nurse Practitioner.

^††^Self-appraisal of overall grade with regard to patient safety in respondent’s unit or department.

Cronbach’s α for each sub-dimension in Japan, Taiwan, and the U.S. was 0.47–0.88, 0.26–0.83, and 0.61–0.87, respectively (Additional file 1).

The mean total and percent positive scores based on sub-dimension are listed in Table 3. The mean score differences among Japan, Taiwan, and the U.S. are shown in Table 4. Although most pairs exhibited significant differences, only some sub-dimensions had pairs with significant differences according to Cohen’s d. In Japan, ‘Organizational Learning/Continuous Improvement’ received the lowest rating among all 3 countries, and ‘Hospital Management Support for Patient Safety’ received a rating lower than that in the U.S. In Taiwan, ‘Frequency of Events Reported’ received the lowest rating among all 3 countries, and ‘Communication Openness’ received a lower rating than that in the U.S. In the U.S., ‘Staffing’ received the highest rating among all 3 countries.

**Table 3:** Total scores and percent positive scores in each sub-dimension

		**Total Score**^**†**^			**Percent Positive Score**^**††**^		
		M	SD	95% CI	M	SD	95% CI
Frequency of events reported	Japan	11.92	2.97	(11.85–11.99)	0.68	0.41	(0.67–0.69)
	Taiwan	9.33	2.50	(9.28–9.38)	0.33	0.39	(0.32–0.33)
	U.S.	11.14	2.86	(11.13–11.15)	0.61	0.42	(0.61–0.61)
Overall perceptions of patient safety	Japan	13.83	2.51	(13.77–13.89)	0.53	0.35	(0.52–0.54)
	Taiwan	13.45	1.93	(13.41–13.49)	0.52	0.28	(0.51–0.52)
	U.S.	14.34	3.14	(14.33–14.36)	0.63	0.34	(0.63–0.63)
Supervisor/manager expectations and actions promoting safety	Japan	14.68	2.56	(14.62–14.74)	0.62	0.31	(0.62–0.63)
	Taiwan	14.57	2.21	(14.52–14.61)	0.65	0.33	(0.64–0.65)
	U.S.	15.43	3.19	(15.42–15.44)	0.74	0.33	(0.74–0.74)
Organizational learning/continuous improvement	Japan	10.44	1.85	(10.39–10.48)	0.55	0.35	(0.54–0.56)
	Taiwan	11.68	1.44	(11.66–11.71)	0.81	0.30	(0.80–0.81)
	U.S.	11.32	2.03	(11.31–11.33)	0.72	0.34	(0.72–0.72)
Teamwork within hospital units	Japan	14.91	2.65	(14.85–14.97)	0.70	0.34	(0.70–0.71)
	Taiwan	15.50	2.27	(15.46–15.55)	0.79	0.30	(0.79–0.80)
	U.S.	15.68	3.12	(15.67–15.69)	0.79	0.30	(0.79–0.79)
Communication openness	Japan	10.38	2.23	(10.33–10.44)	0.49	0.37	(0.48–0.50)
	Taiwan	9.70	1.90	(9.66–9.74)	0.38	0.35	(0.37–0.39)
	U.S.	10.96	2.41	(10.95–10.97)	0.61	0.37	(0.61–0.61)
Feedback and communication about error	Japan	10.73	2.34	(10.67–10.78)	0.53	0.39	(0.52–0.54)
	Taiwan	10.16	1.86	(10.12–10.20)	0.44	0.33	(0.44–0.45)
	U.S.	11.20	2.52	(11.19–11.21)	0.63	0.38	(0.63–0.63)
Nonpunitive response to error	Japan	9.63	2.34	(9.58–9.69)	0.43	0.37	(0.42–0.44)
	Taiwan	8.78	1.92	(8.74–8.82)	0.29	0.31	(0.29–0.30)
	U.S.	9.42	2.75	(9.41–9.43)	0.42	0.39	(0.42–0.42)
Staffing	Japan	12.16	2.63	(12.10–12.23)	0.37	0.27	(0.36–0.38)
	Taiwan	12.15	2.57	(12.10–12.20)	0.36	0.31	(0.36–0.37)
	U.S.	13.55	3.04	(13.54–13.56)	0.54	0.33	(0.54–0.54)
Hospital management support for patient safety	Japan	10.24	1.98	(10.20–10.29)	0.52	0.37	(0.51–0.52)
	Taiwan	10.57	1.81	(10.53–10.61)	0.58	0.36	(0.58–0.59)
	U.S.	11.18	2.48	(11.17–11.18)	0.70	0.37	(0.70–0.70)
Teamwork across hospital units	Japan	13.12	2.49	(13.06–13.18)	0.44	0.35	(0.43–0.45)
	Taiwan	13.69	2.34	(13.64–13.73)	0.51	0.37	(0.50–0.51)
	U.S.	13.66	3.04	(13.65–13.67)	0.55	0.37	(0.55–0.55)
Hospital handoffs and transitions	Japan	12.61	2.52	(12.55–12.68)	0.35	0.36	(0.35–0.36)
	Taiwan	12.67	2.52	(12.62–12.72)	0.39	0.36	(0.39–0.40)
	U.S.	12.67	3.21	(12.66–12.68)	0.41	0.39	(0.41–0.41)

M, mean; SD, standard deviation; CI, confidence interval.

^†^Calculated as the sum of scores for each item within the same sub-dimension.

^††^Mean percentage of positive responses calculated according to AHRQ instructions for every respondent.

**Table 4:** Comparison of total scores and percent positive scores from each sub-dimension across Japan, Taiwan and the U.S.

			**Comparisons of Total Scores**					**Comparisons of Percent Positive Scores**				
	Country1	Country2	Difference of mean score	Cohen's *d*		*P*	95% CI	Difference of mean score	Cohen's *d*		*P*	95% CI
			(Country1-Country2)					(Country1-Country2)				
Frequency of events reported	Japan	Taiwan	2.59	0.96	^†^	<0.01	(2.48–2.69)	0.35	0.88	^†^	<0.01	(0.34–0.37)
	Japan	U.S.	0.78	0.27		<0.01	(0.70–0.86)	0.07	0.17		<0.01	(0.06–0.08)
	Taiwan	U.S.	−1.81	−0.63	^†^	<0.01	(−1.87–−1.74)	−0.28	−0.68	^†^	<0.01	(−0.29–−0.27)
Overall perceptions of patient safety	Japan	Taiwan	0.38	0.17		<0.01	(0.26–0.49)	0.02	0.05		<0.01	(0.00–0.03)
	Japan	U.S.	−0.51	−0.16		<0.01	(−0.61–−0.42)	−0.10	−0.29		<0.01	(−0.11–−0.09)
	Taiwan	U.S.	−0.89	−0.29		<0.01	(−0.97–−0.82)	−0.12	−0.34		<0.01	(−0.12–−0.11)
Supervisor/manager expectations and actions promoting safety	Japan	Taiwan	0.11	0.05		0.07	(−0.01–0.23)	−0.02	−0.07		<0.01	(−0.03–−0.01)
	Japan	U.S.	−0.75	−0.24		<0.01	(−0.84–−0.66)	−0.11	−0.35		<0.01	(−0.12–−0.10)
	Taiwan	U.S.	−0.86	−0.27		<0.01	(−0.94–−0.79)	−0.09	−0.28		<0.01	(−0.10–−0.08)
Organizational learning—continuous improvement	Japan	Taiwan	−1.25	−0.77	^†^	<0.01	(−1.32–−1.17)	−0.26	−0.80	^†^	<0.01	(−0.27–−0.24)
	Japan	U.S.	−0.88	−0.44		<0.01	(−0.94–−0.83)	−0.17	−0.50	^†^	<0.01	(−0.18–−0.16)
	Taiwan	U.S.	0.36	0.18		<0.01	(0.32–0.41)	0.09	0.26		<0.01	(0.08–0.10)
Teamwork within hospital units	Japan	Taiwan	−0.59	−0.24		<0.01	(−0.71–−0.48)	−0.09	−0.27		<0.01	(−0.10–−0.08)
	Japan	U.S.	−0.77	−0.25		<0.01	(−0.85–−0.68)	−0.09	−0.28		<0.01	(−0.09–−0.08)
	Taiwan	U.S.	−0.17	−0.06		<0.01	(−0.25–−0.10)	0.00	0.00		0.99	(−0.01–0.01)
Communication openness	Japan	Taiwan	0.68	0.33		<0.01	(0.59–0.77)	0.11	0.32		<0.01	(0.10–0.13)
	Japan	U.S.	−0.58	−0.24		<0.01	(−0.65–−0.51)	−0.12	−0.32		<0.01	(−0.13–−0.11)
	Taiwan	U.S.	−1.26	−0.53	^†^	<0.01	(−1.32–−1.21)	−0.23	−0.63	^†^	<0.01	(−0.24–−0.22)
Feedback and communication about error	Japan	Taiwan	0.57	0.27		<0.01	(0.47–0.66)	0.09	0.24		<0.01	(0.07–0.10)
	Japan	U.S.	−0.47	−0.19		<0.01	(−0.54–−0.40)	−0.10	−0.25		<0.01	(−0.11–−0.09)
	Taiwan	U.S.	−1.03	−0.41		<0.01	(−1.09–−0.97)	−0.18	−0.48		<0.01	(−0.19–−0.17)
Nonpunitive response to error	Japan	Taiwan	0.85	0.41		<0.01	(0.75–0.95)	0.13	0.40		<0.01	(0.12–0.15)
	Japan	U.S.	0.21	0.08		<0.01	(0.13–0.29)	0.00	0.01		0.59	(−0.01–0.02)
	Taiwan	U.S.	−0.64	−0.24		<0.01	(−0.71–−0.58)	−0.13	−0.33		<0.01	(−0.14–−0.12)
Staffing	Japan	Taiwan	0.01	0.01		0.96	(−0.10–0.13)	0.01	0.02		0.39	(−0.01–0.02)
	Japan	U.S.	−1.39	−0.46		<0.01	(−1.48–−1.30)	−0.17	−0.52	^†^	<0.01	(−0.18–−0.16)
	Taiwan	U.S.	−1.41	−0.46		<0.01	(−1.48–−1.33)	−0.18	−0.54	^†^	<0.01	(−0.18–−0.17)
Hospital management support for patient safety	Japan	Taiwan	−0.32	−0.17		<0.01	(−0.42–−0.23)	−0.07	−0.19		<0.01	(−0.08–−0.06)
	Japan	U.S.	−0.93	−0.38		<0.01	(−1.00–−0.86)	−0.19	−0.51	^†^	<0.01	(−0.20–−0.18)
	Taiwan	U.S.	−0.61	−0.25		<0.01	(−0.67–−0.55)	−0.12	−0.32		<0.01	(−0.13–−0.11)
Teamwork across hospital units	Japan	Taiwan	−0.56	−0.23		<0.01	(−0.68–−0.45)	−0.07	−0.19		<0.01	(−0.08–−0.05)
	Japan	U.S.	−0.54	−0.18		<0.01	(−0.63–−0.45)	−0.11	−0.29		<0.01	(−0.12–−0.10)
	Taiwan	U.S.	0.03	0.01		0.68	(−0.05–0.10)	−0.04	−0.11		<0.01	(−0.05–−0.03)
Hospital handoffs and transitions	Japan	Taiwan	−0.05	−0.02		0.55	(−0.18–0.07)	−0.04	−0.11		<0.01	(−0.05–−0.02)
	Japan	U.S.	−0.06	−0.02		0.35	(−0.15–0.04)	−0.05	−0.14		<0.01	(−0.07–−0.04)
	Taiwan	U.S.	0.00	0.00		1.00	(−0.08–0.07)	−0.02	−0.04		<0.01	(−0.03–−0.01)

P, according to Tukey's honestly significant difference (HSD) test; CI, confidence interval.

^†^Cohen's d > |0.5|.

**Figure 1:**
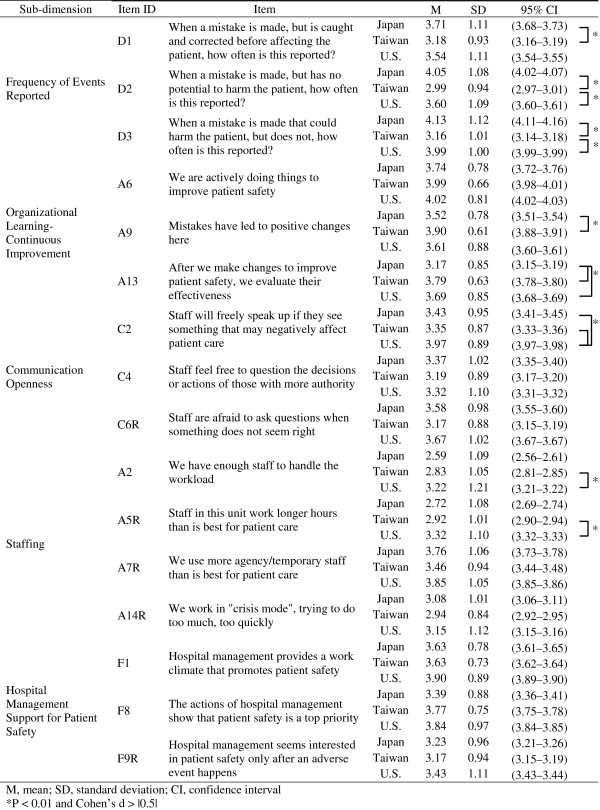
The scores of each item in the 5 sub-dimensions.

The mean score for each item under each of the 5 sub-dimensions is shown in Figure 1, and the mean score differences among Japan, Taiwan, and the U.S. are shown in Additional file 2. In Taiwan, the scores for most items under sub-dimension ‘Frequency of Events Reported’ were significantly lower than the scores in other countries. In Japan, the mean score of A13, the item on evaluations of improvement effect, received the lowest rating among all 3 countries. In the U.S., the mean score of C2, the item regarding staff capacity to point out poor care of patients by other staff, received the highest rating among all 3 countries.

## Discussion

With regard to PSC outcome indicators, Japan had the highest proportion of respondents who had experienced event-reporting during the past 12 months, and the Patient Safety Grade was the highest in the U.S. Regarding PSC characteristics, an attitude of continuous improvement in Japan and the reporting of near-miss events in Taiwan were evaluated low among all 3 countries. In the U.S., staffing received a high rating.

### PSC characteristics in each country

For continuous improvement of safety and quality, organizations need to construct a Deming Cycle or analyse the course of adverse events to mitigate recurrence [17]. However, in Japan, the sub-dimension ‘Organizational Learning/Continuous Improvement’ was rated low due to the lack of evaluation on the effectiveness of changes implemented to improve patient safety. In the past, these types of improvement assessments were absent in most U.S. hospitals as well [18]. The evaluation systems used for improvement, such as monitoring or benchmarking, require quality indicators. These evaluations require management and institutional support for patient safety because related activities may be taxing in the work place and may minimize productivity. Therefore, in Japan, the lack of ‘Organizational Learning/Continuous Improvement’ and ‘Hospital Management Support for Patient Safety’ may be related.

Under-reporting of adverse events in healthcare settings is a common problem throughout the world [19]. Several studies have revealed that healthcare workers often do not report adverse events for fear of management reaction, blame, or being deemed incompetent. A culture of non-punitive response to error is required in healthcare settings [19-21]. In Taiwan, the attitude towards reporting near-miss events, which is reflected in the evaluation of sub-dimension ‘Frequency of Events Reported’, received the lowest rating among all 3 countries. The sub-dimension ‘Non-punitive Response to Error’ also received a low rating in Taiwan. In Chinese societies, authoritarianism is considered an important feature of leadership; and higher ranking individuals tend to speak poorly of subordinates in an effort to maintain their own dominance [22]. In addition, ‘face-saving’ is also considered an important tenet of interpersonal interactions in East Asian societies, and healthcare workers in Taiwan may fear to ‘lose face’ by reporting events [21,22]. This phenomenon may explain the lack of ‘Communication Openness’ in Taiwan; this sub-dimension includes several items that explore reporting possibilities with regard to a co-worker’s faults. Taiwanese individuals may be more likely to avoid reporting the faults of co-workers because intra-group harmony and in-group solidarity are emphasized in East Asian societies, and saving face of a co-worker is important to Taiwanese healthcare workers [21,22]. Those cultures in Taiwan also may affect attitudes toward reporting of near-miss events. Japanese healthcare workers were more likely to report near-miss events, despite Japan being an East Asian country. Japanese healthcare workers may have become familiar with event-reporting systems because nearly a decade has passed since all Japanese hospitals established an in-house, legally bound event-reporting system in 2002.

In the U.S., the sub-dimension ‘Staffing’ received high ratings because of the higher number of healthcare workers in U.S. hospitals than that in Japan and Taiwan. According to ‘OECD Health Data 2010’ and national statistics of Taiwan, the number of nurses per bed was 5 times higher (3.4) than that in Japan (0.7), and 4 times higher than that in Taiwan (0.8) [23,24]. U.S. hospitals may encourage job-sharing and part-time schedules because the proportion of U.S. respondents who work fewer than 40 hours per week was significantly higher than that in Japan or Taiwan. By varying the number of part-time workers, U.S. hospitals may be better positioned to adapt the number of healthcare workers to sudden changes in demand of manpower. In U.S. hospitals, temporary nurses, who are identified as ‘agency nurses’ or ‘travel nurses’ are hired [25]. In the contrast, most hired nurses in the Japanese and Taiwanese hospitals are permanent staff.

Japanese healthcare workers had more event-reporting experience than Taiwan or the U.S. A possible explanation may lie in the high rating that Japan received for the sub-dimension ‘Non-punitive Response to Error’; however, the U.S. exhibited a rating for this sub-dimension similar to that of Japan. Other U.S. factors, such as self-protection against legal action or job loss, may contribute to the smaller proportion of U.S. respondents who noted experiences of event-reporting. The small proportion of nurses in the U.S. respondent pool also may have affected this rating because, as is widely known, most event reports originate from nurses [26]. In addition, the definitions or perceptions of reportable events may differ across the 3 countries.

### Challenges associated with PSC comparison

In a cross-cultural study, the differences among societies were shown to be influenced by several factors, such as study design, traditional values, or socioeconomic status [22]. The first problem is translation accuracy of the questionnaire in the context of culture [22]. Most sub-dimensions, except ‘Overall Perceptions of Patient Safety’ in Taiwan, exhibit sufficient internal consistency. Relatively low levels of internal consistency related to ‘Staffing’ were common in all 3 countries, and similar problems were pointed out in other studies conducted outside the U.S [3,6,13]. The problem may not be translation but rather the items that comprise the sub-dimension ‘Staffing’. Employment system characteristics of each country should be reflected in these items.

The second problem pertains to target population representation [27]. Taiwanese hospitals were selected according to stratified sampling techniques, but Japanese and U.S. hospitals participated voluntarily. The voluntary hospitals may be more aware of PSC, and thereby, the respondents in Japan and the U S may have better PSC compared with respondents in Taiwan. The U.S. response rate was lower than the response rate in Japan and Taiwan, and the characteristics of non-respondents were unknown [28].

The third problem is the central tendency response pattern [29]. For example, with regard to Patient Safety Grade, Japanese and Taiwanese respondents were more likely to choose ‘Acceptable’, which was the central option of the 5-point Likert scale. This selection pattern may lead to a skewed proportion of ‘Excellent’ and ‘Very Good’ responses in Japan and Taiwan that are lower than those in the U.S. In East Asian societies, people may be more inclined to offer ambiguous opinions because they fear that clear or extreme opinions occasionally may yield adverse effects on intra-group harmony and in-group solidarity [21,22]. In this study, the percent positive score results were similar to total score results, which suggests that the effect of this central tendency was small.

The fourth problem is that the comparison was based on subjective evaluations of PSC. Subjective evaluation often is inconsistent with objective evaluations because it is influenced by multiple factors, including internal predispositions of respondents [30]. To identify the impacts of different PSC on objective outcomes of patient safety, we need tools such as Patient Safety Indicators. PSC in each country should be adjusted according to objective data with regard to patient safety.

## Conclusions

Healthcare workers in the U.S. were likely to evaluate their PSC as higher than that in Japan or Taiwan. The attitudes towards continuous improvement in Japanese healthcare workers and the reporting of near-miss events in Taiwanese healthcare workers were evaluated as low. The results of this study suggest that PSC varies among different countries, and the effective intervention to improve PSC should be developed with focus on the cultural background of the country. Further investigations with improved methodology and a common protocol will be required for accurate comparison of PSC among countries.

## Competing interests

The authors declare no competing interests.

## Authors’ contributions

SF participated in study design and coordination, collected data, preformed statistical analysis, and drafted the manuscript. KS participated in study design and sequence alignment. SI collected data and performed statistical analysis. YW participated in sequence alignment. HCC provided data from Taiwan. TH conceived the study, participated in its design, and helped draft the manuscript. All authors read and approved the final manuscript.

## Pre-publication history

The pre-publication history for this paper can be accessed here:

http://www.biomedcentral.com/1472-6963/13/20/prepub

## Supplementary Material

Additional file 1 Internal consistency of each sub-dimension of HSOPS in Japan, Taiwan and the U.S. Click here for file

Additional file 2 Comparisons of scores by items in 5 sub-dimensions among Japan, Taiwan and the U.S. Click here for file
