# Supplementary material for: The characteristics of patient safety culture in Japan, Taiwan and the United States
Source: BMC Health Serv Res. 2013 Jan 14;13:20. doi: 10.1186/1472-6963-13-20 (PMC3626628; doi:10.1186/1472-6963-13-20)
Supplement: Additional file 2 — Comparisons of scores by items in 5 sub-dimensions among Japan, Taiwan and the U.S. [file 1472-6963-13-20-S2.doc]

### Additional file 2 – Comparisons of scores by items in 5 sub-dimensions among Japan, Taiwan and the U.S.

|  |  |  | Country1 | Country2 | Remainder of mean score (Country1-Country2) | Cohen's d |  | *P* | 95% CI |
| --- | --- | --- | --- | --- | --- | --- | --- | --- | --- |
| Frequency of Events Reported | D1 | When a mistake is made, but is caught and corrected before affecting the patient, how often is this reported? | Japan | Taiwan | 0.53 | 0.53 | † | <0.01 | (0.49‐0.57) |
|  |  |  | Japan | U.S. | 0.16 | 0.15 |  | <0.01 | (0.13‐0.19) |
|  |  |  | Taiwan | U.S. | -0.37 | -0.33 |  | <0.01 | (-0.39‐-0.34) |
|  | D2 | When a mistake is made, but has no potential to harm the patient, how often is this reported? | Japan | Taiwan | 1.06 | 1.06 | † | <0.01 | (1.02‐1.10) |
|  |  |  | Japan | U.S. | 0.44 | 0.41 |  | <0.01 | (0.41‐0.48) |
|  |  |  | Taiwan | U.S. | -0.61 | -0.56 | † | <0.01 | (-0.64‐-0.58) |
|  | D3 | When a mistake is made that could harm the patient, but does not, how often is this reported? | Japan | Taiwan | 0.97 | 0.91 | † | <0.01 | (0.93‐1.01) |
|  |  |  | Japan | U.S. | 0.14 | 0.14 |  | <0.01 | (0.11‐0.17) |
|  |  |  | Taiwan | U.S. | -0.83 | -0.82 | † | <0.01 | (-0.85‐-0.80) |
| Organizational Learning-Continuous Improvement | A6 | We are actively doing things to improve patient safety | Japan | Taiwan | -0.25 | -0.35 |  | <0.01 | (-0.28‐-0.22) |
|  |  |  | Japan | U.S. | -0.28 | -0.35 |  | <0.01 | (-0.30‐-0.26) |
|  |  |  | Taiwan | U.S. | -0.03 | -0.04 |  | <0.01 | (-0.05‐-0.01) |
|  | A9 | Mistakes have led to positive changes here | Japan | Taiwan | -0.37 | -0.54 | † | <0.01 | (-0.40‐-0.34) |
|  |  |  | Japan | U.S. | -0.08 | -0.09 |  | <0.01 | (-0.11‐-0.06) |
|  |  |  | Taiwan | U.S. | 0.29 | 0.33 |  | <0.01 | (0.27‐0.31) |
|  | A13 | After we make changes to improve patient safety, we evaluate their effectiveness | Japan | Taiwan | -0.62 | -0.85 | † | <0.01 | (-0.65‐-0.59) |
|  |  |  | Japan | U.S. | -0.52 | -0.61 | † | <0.01 | (-0.54‐-0.49) |
|  |  |  | Taiwan | U.S. | 0.10 | 0.12 |  | <0.01 | (0.08‐0.12) |
| Communication Openness | C2 | Staff will freely speak up if they see something that may negatively affect patient care | Japan | Taiwan | 0.08 | 0.09 |  | <0.01 | (0.05‐0.12) |
|  |  |  | Japan | U.S. | -0.54 | -0.61 | † | <0.01 | (-0.57‐-0.52) |
|  |  |  | Taiwan | U.S. | -0.63 | -0.70 | † | <0.01 | (-0.65‐-0.61) |
|  | C4 | Staff feel free to question the decisions or actions of those with more authority | Japan | Taiwan | 0.19 | 0.20 |  | <0.01 | (0.15‐0.23) |
|  |  |  | Japan | U.S. | 0.06 | 0.05 |  | <0.01 | (0.02‐0.09) |
|  |  |  | Taiwan | U.S. | -0.13 | -0.12 |  | <0.01 | (-0.16‐-0.11) |
|  | C6R | Staff are afraid to ask questions when something does not seem right | Japan | Taiwan | 0.41 | 0.44 |  | <0.01 | (0.37‐0.44) |
|  |  |  | Japan | U.S. | -0.09 | -0.09 |  | <0.01 | (-0.12‐-0.07) |
|  |  |  | Taiwan | U.S. | -0.50 | -0.49 |  | <0.01 | (-0.53‐-0.48) |
| Staffing | A2 | We have enough staff to handle the workload | Japan | Taiwan | -0.24 | -0.23 |  | <0.01 | (-0.29‐-0.20) |
|  |  |  | Taiwan | U.S. | -0.63 | -0.52 | † | <0.01 | (-0.66‐-0.59) |
|  |  |  | U.S. | U.S. | -0.39 | -0.32 |  | <0.01 | (-0.42‐-0.36) |
|  | A5R | Staff in this unit work longer hours than is best for patient care | Japan | Taiwan | -0.20 | -0.19 |  | <0.01 | (-0.24‐-0.16) |
|  |  |  | Taiwan | U.S. | -0.60 | -0.55 | † | <0.01 | (-0.63‐-0.57) |
|  |  |  | U.S. | U.S. | -0.40 | -0.37 |  | <0.01 | (-0.43‐-0.38) |
|  | A7R | We use more agency/temporary staff than is best for patient care | Japan | Taiwan | 0.30 | 0.30 |  | <0.01 | (0.26‐0.34) |
|  |  |  | Taiwan | U.S. | -0.09 | -0.09 |  | <0.01 | (-0.13‐-0.06) |
|  |  |  | U.S. | U.S. | -0.39 | -0.38 |  | <0.01 | (-0.42‐-0.37) |
|  | A14R | We work in "crisis mode", trying to do too much, too quickly | Japan | Taiwan | 0.15 | 0.16 |  | <0.01 | (0.10‐0.19) |
|  |  |  | Taiwan | U.S. | -0.07 | -0.06 |  | <0.01 | (-0.10‐-0.04) |
|  |  |  | U.S. | U.S. | -0.22 | -0.19 |  | <0.01 | (-0.24‐-0.19) |
| Hospital Management Support for Patient Safety | F1 | Hospital management provides a work climate that promotes patient safety | Japan | Taiwan | 0.00 | 0.00 |  | 1.00 | (-0.03‐0.03) |
|  |  |  | Japan | U.S. | -0.27 | -0.30 |  | <0.01 | (-0.29‐-0.24) |
|  |  |  | Taiwan | U.S. | -0.27 | -0.30 |  | <0.01 | (-0.29‐-0.25) |
|  | F8 | The actions of hospital management show that patient safety is a top priority | Japan | Taiwan | -0.38 | -0.47 |  | <0.01 | (-0.42‐-0.35) |
|  |  |  | Japan | U.S. | -0.46 | -0.47 |  | <0.01 | (-0.48‐-0.43) |
|  |  |  | Taiwan | U.S. | -0.08 | -0.08 |  | <0.01 | (-0.10‐-0.05) |
|  | F9R | Hospital management seems interested in patient safety only after an adverse event happens | Japan | Taiwan | 0.06 | 0.07 |  | <0.01 | (0.02‐0.11) |
|  |  |  | Japan | U.S. | -0.20 | -0.18 |  | <0.01 | (-0.23‐-0.17) |
|  |  |  | Taiwan | U.S. | -0.26 | -0.24 |  | <0.01 | (-0.29‐-0.24) |
| † : Cohen's d > ｜0.5｜, P: P value of Tukey's HSD test, CI: Confidence interval | | | | | | | | | |
